# Outcomes of cardiac resynchronization therapy (CRT) in cardiac sarcoidosis patients with a range of ejection fractions

**DOI:** 10.1002/ehf2.15113

**Published:** 2024-10-17

**Authors:** Alexander Liu, Raheel Ahmed, Mansimran Singh Dulay, Joseph Okafor, Alessia Azzu, Kamleshun Ramphul, Rui Shi, Gerald Ballo, John Arun Baksi, Kshama Wechalekar, Rajdeep Khattar, Peter Collins, Athol Umfrey Wells, Vasilis Kouranos, Rakesh Sharma

**Affiliations:** ^1^ Royal Brompton and Harefield Hospitals, part of Guy's and St Thomas' NHS Foundation Trust London UK; ^2^ National Heart and Lung Institute Imperial College London London UK; ^3^ King's College London London UK; ^4^ Independent Researcher Triolet Mauritius

**Keywords:** cardiac resynchronization therapy, cardiac sarcoidosis, outcomes

## Abstract

**Aims:**

In cardiac sarcoidosis (CS) patients, the benefit of cardiac resynchronization therapy (CRT) remains unclear. We sought to assess the short‐term and long‐term effects of CRT in CS patients with a range of left ventricular (LV) ejection fractions (LVEFs).

**Methods:**

Consecutive CS patients with heart failure with reduced ejection fraction (HFrEF; LVEF ≤ 40%), mildly reduced ejection fraction (HFmrEF; LVEF 41%–49%) and preserved ejection fraction (HFpEF; LVEF ≥ 50) treated with CRT under the care of a tertiary UK centre between 2008 and 2023 were reviewed. CRT response was defined by >5% improvement in serial LVEF. The primary endpoint was a composite of all‐cause mortality, cardiac transplantation or unplanned hospitalization for decompensated heart failure. The secondary endpoint included ventricular arrhythmic events.

**Results:**

Of the 100 patients enrolled (age 58 ± 10 years; 71% male), 63 had HFrEF, 17 had HFmrEF and 20 had HFpEF. After short‐term follow‐up (9.8 ± 5.4 months), HFrEF patients demonstrated significant LVEF response (*P* < 0.01). On Kaplan–Meier analysis (follow‐up 38 ± 32 months), HFrEF non‐responders had significantly worse event‐free survival compared with HFrEF responders for the primary (*P* < 0.001) and secondary (*P* = 0.001) endpoints. Despite short‐term LV function improvement, CRT responders still had worse event‐free survival compared with HFmrEF/HFpEF patients for the primary endpoint (*P* < 0.001). On multivariable Cox analysis, age [hazard ratio (HR) 1.05, 95% confidence interval (CI) 1.01–1.10, *P* = 0.008] and HFrEF CRT non‐response (HR 12.33, 95% CI 2.45–61.87, *P* = 0.002) were associated with the primary endpoint.

**Conclusions:**

In CS patients with HFrEF, CRT response is associated with a better long‐term prognosis than non‐response. However, HFrEF CRT responders still have worse long‐term prognosis than HFmrEF/HFpEF patients.

## Graphical abstract

Outcomes of cardiac resynchronization therapy in cardiac sarcoidosis patients with a range of left ventricular ejection fractions.

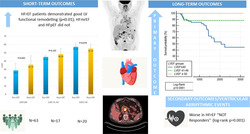

## Introduction

Sarcoidosis is a chronic multi‐system inflammatory condition characterized by the formation of non‐caseating granulomas in affected tissues.^1^ Whilst pulmonary involvement is commonest, occurring in around 90% of cases,^2^ cardiac involvement is also known to be present in up to a quarter of sarcoidosis patients based on contemporary imaging data and autopsy studies.^3^, ^4^, ^5^ Patients with cardiac sarcoidosis (CS) can present with heart failure, ventricular arrhythmias and cardiac conduction defects, for instance, high‐grade atrioventricular heart block (AVB) and left bundle branch block.^6^, ^7^, ^8^ A small proportion of CS patients can also present with sudden cardiac death (SCD).^6^ Therefore, effective management of heart failure and potentially life‐threatening ventricular arrhythmias in CS patients is a high clinical priority.^9^, ^10^, ^11^

In patients with heart failure with reduced ejection fraction (HFrEF) and either left/right bundle branch block or chronic right ventricular (RV) pacing, cardiac resynchronization therapy (CRT) is indicated to promote cardiac functional recovery and improve long‐term prognosis.^12^, ^13^ CRT is not indicated in patients with heart failure with mildly reduced ejection fraction (HFmrEF) or preserved ejection fraction (HFpEF).^12^, ^13^ Whilst these approaches have been adopted by international guidelines for non‐CS indications, the use of CRT in CS patients with HFrEF has remained extrapolatory and controversial.^14^, ^15^, ^16^, ^17^, ^18^ In such patients, CRT implantation has generated mixed results in terms of short‐term improvements in left ventricular (LV) systolic function,^14^, ^15^, ^16^, ^17^, ^18^ with poor long‐term outcomes.^14^, ^15^, ^16^, ^17^, ^18^ However, the existing studies on CRT patients have a relatively small sample size^14^, ^15^, ^16^, ^17^, ^18^ and with potentially a variable degree of CS diagnostic confidence.^19^ The true effect of CRT in CS patients in terms of short‐term cardiac remodelling and long‐term prognosis remains unclear.^20^

There is currently an important knowledge gap in relation to the indication of CRT in CS patients without significant LV systolic dysfunction. Indeed, in these patients, CRT is not expected to lead to marked LV functional recovery but is often considered in the context of CS with high‐grade AVB.^21^ Do they do as well as CS patients with HFrEF in terms of short‐term cardiac functional recovery? Are there predictors beyond LV ejection fraction (LVEF) that can predict clinical outcomes in CS patients undergoing CRT implantation? The answers to these questions may offer mechanistic insights into the role of CRT in CS patients.

In this study, we assessed the short‐term and long‐term outcomes in a cohort of contemporary CS patients with a wide range of LVEFs.

## Methods

### Study population

All adult CS patients referred to the Royal Brompton Hospital (London, UK) between November 2008 and March 2023 were retrospectively reviewed. A confirmed or suspected CS diagnosis was given following multi‐disciplinary team (MDT) discussion, in accordance with the Heart Rhythm Society (HRS) recommendations as previously described.^3^, ^4^ Patients who underwent a CRT implantation [either CRT—pacemaker (CRT‐P) or CRT—defibrillator (CRT‐D)] were considered eligible for this study. Patients requiring a CRT upgrade due to a new CS diagnosis and/or deterioration of their LV function were also included.

The baseline LVEF at the time of CRT implantation was used to divide the CS patient cohort into three groups: HFpEF (LVEF ≥ 50%), HFmrEF (LVEF 41%–49%) and HFrEF (LVEF ≤ 40%).^12^ Heart failure medical therapy was optimized by experienced heart failure specialist nurses in accordance with renal function, blood pressure and side effects.

Patients with coexisting significant ischaemic heart disease, an alternative cardiomyopathic process, significant valvular heart disease or those with incomplete follow‐up data were excluded.

### CRT implantation

Patients underwent routine clinical CRT implantations using standard techniques.^13^ All patients received perioperative standard antibiotics and underwent defibrillation threshold testing at the time of implantation unless contraindicated. Patients underwent post‐implantation wound check and device interrogation at 4 weeks, with regular pacing clinic assessments, including electrocardiogram (ECG) recordings and device interrogations, thereafter. Patients who underwent CRT‐D were also followed up with a remote monitoring system to check for ventricular arrhythmias. The zones for detecting ventricular tachycardia (VT) were set at 150 b.p.m. in a primary prevention device, 180 b.p.m. in a secondary prevention device and 220 b.p.m. for detecting ventricular fibrillation. Device programming was performed at the discretion of the implanting cardiologist.

### Clinical data and follow‐up

All patient clinical and follow‐up data were collected by reviewing the hospital electronic patient records or by contacting the referring physician or general practitioner.

In the short term, patients underwent transthoracic echocardiography (TTE) at their baseline CS diagnosis and ≥6 months afterwards. LVEF was estimated using the Simpson biplane method.^22^ Patients were deemed CRT responders if they had ≥5% improvement in LVEF on serial TTE, as previously described.^17^, ^20^ Other TTE parameters collected include LV internal diameter during systole (LVIDs), LV internal diameter during diastole (LVIDd), LV end‐systolic volume index (LVESVi), LV end‐diastolic volume index (LVEDVi), left atrial volume index (LAVi), RV basal diameter, mitral regurgitation (MR) on a scale of 0–9 (none, trivial, mild, mild to moderate, moderate, moderate to severe, severe, very severe and torrential) and tricuspid annular plane systolic excursion (TAPSE).

In the long term, patients were followed up for clinical events until 1 March 2024. The primary study endpoint was a composite of all‐cause mortality, cardiac transplantation and unplanned hospitalization for acute decompensated heart failure. A secondary endpoint consisted of ventricular arrhythmic events (VAEs), defined as sustained VT (heart rate >150 b.p.m., lasting >30 s as detected on CRT interrogation), and/or aborted SCD, defined as appropriate anti‐tachycardia pacing and/or shock therapy. Clinical ECG was also reviewed whenever available. Echocardiography was performed for clinical reasons and was not systematically available at the long‐term follow‐up time points.

### Ethical approval

The Royal Brompton and Harefield Research Office approved the study and waived informed patient consent given the retrospective nature of the data.

### Statistical analysis

Data were analysed using commercially available software (SPSS, IBM, Vol. 25) and expressed as mean ± standard deviation or medians with inter‐quartile range. Group comparisons were made using Student's *t* test for normally distributed variables, the Mann–Whitney *U* test for non‐normally distributed variables, the chi‐squared test or Fisher's exact test for categorical variables, as appropriate. Differences across three patient subgroups were analysed using the one‐way ANOVA for parametric data and the Kruskal–Wallis test for non‐parametric data. A subgroup analysis was performed to identify responders to CRT therapy. Univariate Cox proportional hazard models were used to assess the association between baseline covariates and primary endpoint [presented as hazard ratios (HRs) and 95% confidence intervals (CIs)]. Time to event was calculated as the period between the CRT implantation and the first event or death. Those not reaching the endpoint were censored at the time of follow‐up (1 March 2024). Age, gender and clinically important variables with *P* < 0.05 on univariate analyses were included in multivariate analysis. Kaplan–Meier survival curves were created for selected independent predictors to assess differences in cumulative event‐free survival. All statistical tests were two‐tailed, and a *P* value of <0.05 was considered statistically significant.

## Results

### Baseline characteristics

Table 1 presents the baseline characteristics of the three cohorts of CS patients with CRT. A total of 100 patients (4 with CRT‐P and 96 with CRT‐D) met the inclusion criteria during the study period (HFrEF: 63; HFmrEF: 17; and HFpEF: 20). The patients (mean age 57.9 ± 10.1 years) were predominantly male (71%) and Caucasian (81%). Four patients had definite CS with a positive endomyocardial biopsy, 52 had probable CS with extra‐cardiac biopsy evidence of sarcoidosis, whilst the remaining 44 patients had a presumed clinical diagnosis of CS after MDT review.^23^ Overall, 74 patients had CS with evidence of extra‐cardiac sarcoidosis, and 26 patients had isolated CS. A total of 35 patients underwent upgrade to CRT [*n* = 27 previously had a permanent pacemaker (PPM) and *n* = 8 previously had an implantable cardioverter defibrillator (ICD)].

**Table 1:** Baseline characteristics.

	LVEF ≤ 40 (*n* = 63)	LVEF 41–49 (*n* = 17)	LVEF ≥ 50 (*n* = 20)	*P* value
Age (years), mean ± SD	57.52 (±10.73)	60.41 (±10.08)	57.10 (±8.19)	0.539
Male (%)	46 (73.0)	12 (70.6)	13 (65.0)	0.788
Caucasian (%)	49 (77.8)	14 (82.4)	18 (90.0)	0.576
Device upgrades (%)	23 (36.5)	4 (23.5)	8 (40.0)	0.531
Defibrillator (%)	59 (93.7)	17 (100)	20 (100)	0.456
Prior VT (%)	14 (22.2)	5 (29.4)	4 (20.0)	0.788
Prior AVB (%)	30 (47.6)	12 (70.6)	16 (80.0)	0.020
Prior HF hospitalization (%)	54 (85.7)	15 (88.2)	12 (60.0)	0.041
SUV_max_ > 2.5 at baseline (%)	35/59 (59.3)	9/16 (56.3)	14/19 (73.7)	0.473
Heart biopsy (%)	4 (6.3)	0 (0)	0 (0)	0.456
Extra‐cardiac biopsy (%)	32 (50.8)	8 (47.1)	12 (60)	0.699
Diabetes (%)	16 (25.4)	4 (23.5)	4 (20.0)	0.944
Hypertension (%)	27 (42.9)	4 (23.5)	10 (50.0)	0.234
Chronic kidney disease (%)	10 (15.9)	0 (0.0)	2 (10.0)	0.242
Atrial fibrillation (%)	17 (27.0)	7 (41.2)	3 (15.0)	0.202
Chronic obstructive pulmonary disease (%)	4 (6.3)	0 (0.0)	0 (0.0)	0.456
Cerebrovascular accident (%)	6 (9.5)	2 (11.8)	0 (0.0)	0.327
Baseline electrocardiogram				
LBBB intrinsic (%)	24 (38.1)	8 (47.1)	6 (30.0)	0.567
RBBB intrinsic (%)	11 (17.5)	5 (29.4)	2 (10.0)	0.343
Paced LBBB (%)	21 (33.3)	2 (11.8)	6 (30.0)	0.219
Paced RBBB (%)	3 (4.8)	1 (5.9)	2 (10.0)	0.712
NVCD (%)	4 (6.3)	1 (5.9)	4 (20)	0.171
QRS duration, mean (±SD)	155.95 (±25.21)	152.78 (±13.44)	147.09 (±22.02)	0.534
Baseline echocardiogram				
LVEF, mean (±SD)	29.00 (±8.58)	44.98 (±2.57)	57.39 (±4.77)	<0.001
EDVi (mL), mean (±SD)	96.65 (±41.89)	59.69 (±14.22)	58.20 (±16.07)	<0.001
ESVi (mL), mean (±SD)	69.17 (±39.55)	32.40 (±8.09)	25.20 (±8.26)	<0.001
Medications at baseline				
ACE inhibitor (%)	29 (46.0)	5 (29.4)	7 (35.0)	0.387
ARB (%)	18 (28.6)	7 (41.2)	10 (50.0)	0.182
ARNI (%)	16 (25.4)	1 (5.9)	1 (5.0)	0.053
Beta‐blocker (%)	54 (85.7)	12 (70.6)	13 (65.0)	0.087
MRA (%)	48 (76.2)	7 (41.2)	7 (35.0)	<0.001
SGLT2 (%)	6 (9.5)	3 (17.6)	3 (15.0)	0.499
Digitalis (%)	3 (4.8)	0 (0.0)	0 (0.0)	1.000
Diuretics (%)	22 (34.9)	3 (17.6)	2 (10.0)	0.058
Statin (%)	9 (14.3)	5 (29.4)	4 (20.0)	0.319
Amiodarone (%)	11 (17.5)	0 (0.0)	2 (10.0)	0.177
Corticosteroids (%)	40 (63.5)	13 (76.5)	14 (70.0)	0.571
Methotrexate (%)	31 (49.2)	7 (41.2)	11 (55.0)	0.703
Hydroxychloroquine (%)	14 (22.2)	1 (5.9)	4 (20.0)	0.309
Anticoagulation (%)	26 (41.3)	7 (41.2)	6 (30.0)	0.653
Device complications				
Major (%)	2 (3.2)	2 (11.8)	2 (10.0)	0.194
Minor (%)	5 (7.9)	2 (11.8)	0 (0.0)	0.325

Abbreviations: ACE, angiotensin‐converting enzyme; ARB, angiotensin receptor blocker; ARNI, angiotensin receptor–neprilysin inhibitor; AVB, atrioventricular block; EDVi, end‐diastolic volume index; ESVi, end‐systolic volume index; LBBB, left bundle branch block; LVEF, left ventricular ejection fraction; MRA, mineralocorticoid receptor antagonist; NVCD, nonspecific ventricular conduction delay; RBBB, right bundle branch block; SD, standard deviation; SGLT2, sodium–glucose transport protein 2; SUV_max_, maximum standardized uptake value; VT, ventricular tachycardia.

Prior to CRT implantation, 58 patients had pre‐existing high‐grade AVB, 23 patients had VAEs, and 81 patients had previously experienced hospitalization due to acute decompensated heart failure. Patients with HFrEF and HFmrEF were significantly more likely to have had a history of decompensated heart failure hospitalization, as compared with HFpEF CS patients (85.7% and 88.2% vs. 60.0%, *P* = 0.04). HFpEF patients were significantly more likely to have a pre‐existing history of high‐grade AVB (80% vs. 47.6% and 70.6%, *P* = 0.02).

At baseline, 58/94 (61.7%) CS patients demonstrated evidence of active myocardial inflammation on fludeoxyglucose‐18 (FDG) positron emission tomography (PET) scans, as defined by a maximum standardized uptake value (SUV_max_) >2.5. Patients in the three LVEF categories had a similar prevalence of myocardial inflammation (Table 1). In terms of baseline immunosuppressive therapy, 67 patients were taking prednisolone, 49 patients were taking methotrexate, and 19 patients were taking hydroxychloroquine. These were not significantly different in patients across the three LVEF categories (Table 1). A greater proportion of HFrEF patients were taking mineralocorticoid receptor antagonist (MRA), as compared with patients in other LVEF ranges (Table 1). There were no significant differences in other guideline‐directed heart failure medical therapies across the LVEF categories (Table 1). The baseline ECG was similar between the three LVEF patient cohorts.

### Short‐term clinical and echocardiographic follow‐up

**Figure 1:**
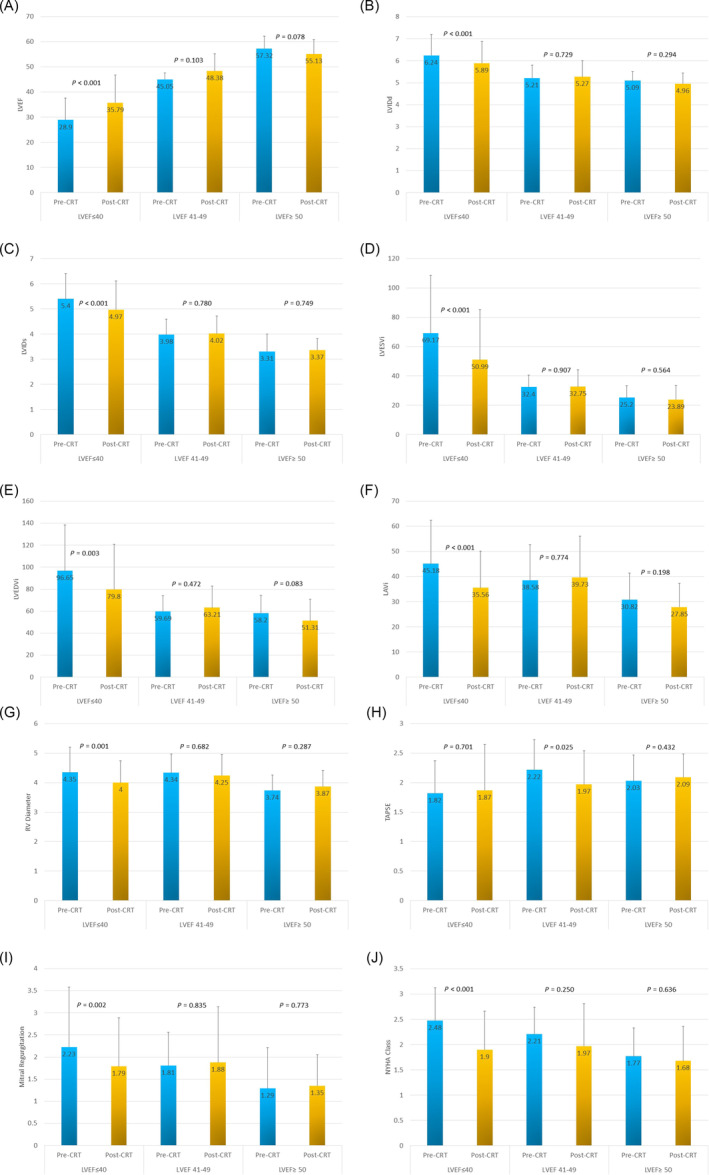
(A) Bar graphs representing change in serial left ventricular ejection fraction (LVEF) after cardiac resynchronization therapy (CRT) in cardiac sarcoidosis patients. (B) Bar graphs representing change in serial left ventricular internal diameter during diastole (LVIDd) after CRT in cardiac sarcoidosis patients. (C) Bar graphs representing change in serial left ventricular internal diameter during systole (LVIDs) after CRT in cardiac sarcoidosis patients. (D) Bar graphs representing change in serial left ventricular end‐systolic volume index (LVESVi) after CRT in cardiac sarcoidosis patients. (E) Bar graphs representing change in serial left ventricular end‐diastolic volume index (LVEDVi) after CRT in cardiac sarcoidosis patients. (F) Bar graphs representing change in serial left atrial volume index (LAVi) after CRT in cardiac sarcoidosis patients. (G) Bar graphs representing change in serial right ventricular (RV) diameter after CRT in cardiac sarcoidosis patients. (H) Bar graphs representing change in serial tricuspid annular plane systolic excursion (TAPSE) after CRT in cardiac sarcoidosis patients. (I) Bar graphs representing change in serial mitral regurgitation after CRT in cardiac sarcoidosis patients. (J) Bar graphs representing change in serial New York Heart Association (NYHA) classification after CRT in cardiac sarcoidosis patients. Numbers inside bars are means, and error bars are standard deviation.

At baseline, compared with HFmrEF and HFpEF patients, HFrEF patients had significantly lower LVEF and greater LV systolic and diastolic indexed volumes (all *P* < 0.001). At least 6 months after CRT implantation (mean 9.76 ± 5.40 months), HFrEF patients demonstrated significant improvements in LVEF, LVIDd, LVIDs, LVEDVi, LVESVi, LAVi, RV diameter, MR and New York Heart Association (NYHA) (all *P* < 0.01). TAPSE did not improve significantly on follow‐up (Figure 1).

During the same short‐term follow‐up, HFmrEF and HFpEF patients demonstrated no significant improvement in TTE parameters. HFrEF had significantly more CRT responders: 43/61 (70.5%) versus 9/16 (56.3%) and 3/18 (16.7%), *P* < 0.001.

In total, six patients had major device complications and seven patients had minor device complications after CRT implantation (HFrEF major, *n* = 2: one device infection and one lead displacement; HFmrEF major, *n* = 2: one lead displacement and one haemothorax; HFpEF, *n* = 2: one device infection and one lead displacement; HFrEF minor, *n* = 5: two wound infections, one large haematoma, one swollen arm and one prolonged sternal rash; and HFmrEF minor, *n* = 2: one wound infection and one haematoma). There was no significant difference in major or minor complication risk between the three cohorts.

### Long‐term follow‐up

**Figure 2:**
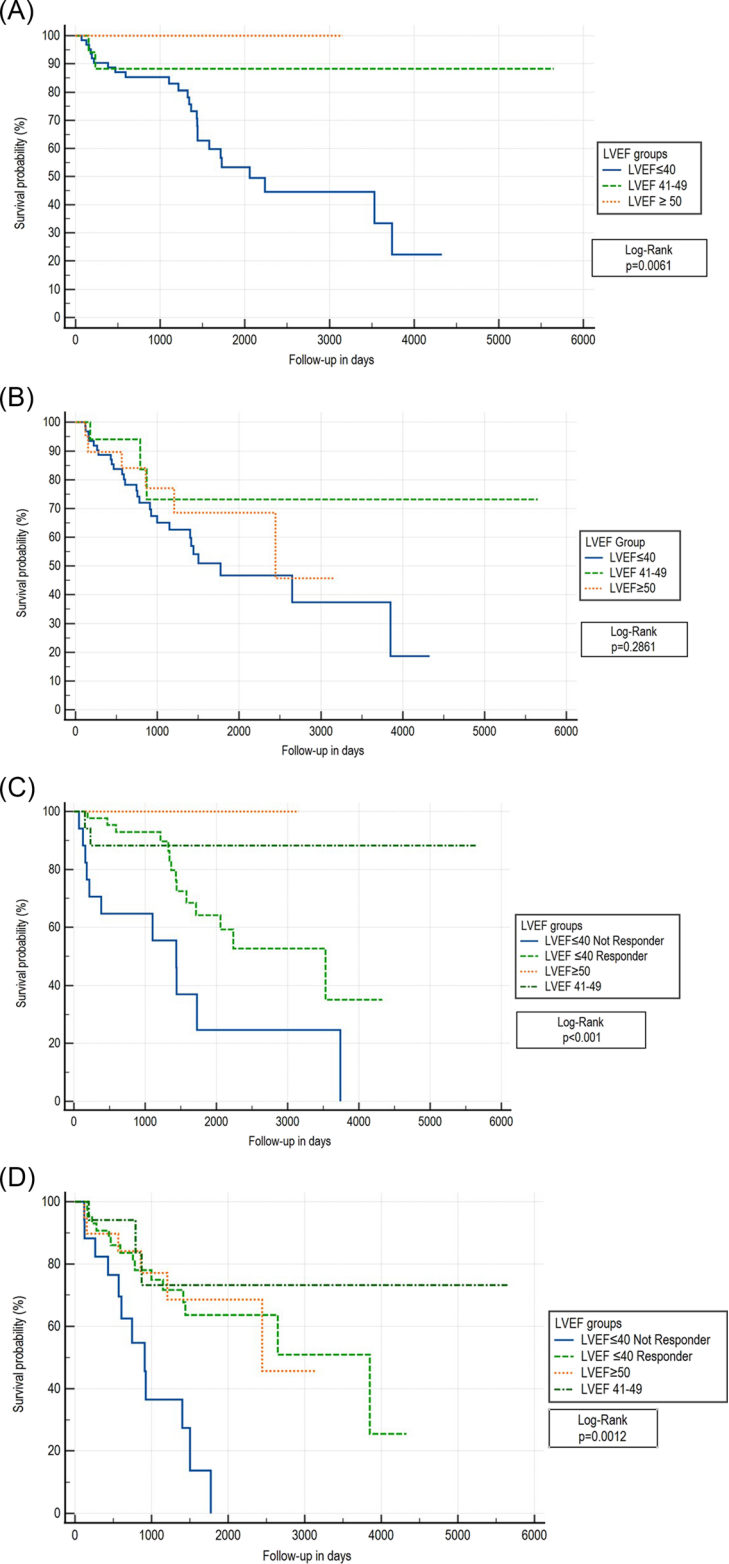
(A) Kaplan–Meier curve for primary composite endpoint in cardiac sarcoidosis (CS) patients with cardiac resynchronization therapy (CRT) with baseline left ventricular ejection fraction (LVEF) ≤40, LVEF 41–49 and LVEF ≥ 50. (B) Kaplan–Meier curve for secondary endpoint in CS patients with CRT with baseline LVEF ≤ 40, LVEF 41–49 and LVEF ≥ 50. (C) Kaplan–Meier curve for primary composite endpoint in CS patients with CRT with baseline LVEF ≤ 40 non‐responder, LVEF ≤ 40 responder, LVEF 41–49 and LVEF ≥ 50. (D) Kaplan–Meier curve for secondary endpoint in CS patients with CRT with baseline LVEF ≤ 40 non‐responder, LVEF ≤ 40 responder, LVEF 41–49 and LVEF ≥ 50.

After a mean follow‐up period of 38.3 ± 32.1 months, the primary endpoint of all‐cause mortality, cardiac transplantation or unplanned hospitalization for acute decompensated heart failure (first event) was reached by 27 patients [HFrEF, *n* = 25 (39.7%); HFmrEF, *n* = 2 (11.8%); and HFpEF, *n* = 0 (0%); log‐rank *P* = 0.006 (Figure 2A)]. Overall, there were 19 deaths [HFrEF: 18 (28.6%); HFmrEF: 1 (5.9%); and HFpEF: 0 (0%)], 4 cardiac transplantations [HFrEF: 4 (6.4%); HFmrEF: 0 (0%); and HFpEF: 0 (0%)] and 17 hospitalizations for acute decompensated heart failure [HFrEF: 16 (25.4%); HFmrEF: 1 (5.9%); and HFpEF: 0 (0%)] during the follow‐up period.

On univariable analysis, age (HR 1.041, 95% CI 1.002–1.083, *P* = 0.040), HFrEF CRT responders (HR 4.946, 95% CI 1.123–21.790, *P* = 0.035) and HFrEF non‐responders (HR 15.150, 95% CI 3.342–68.682, *P* < 0.001) were associated with the primary endpoint.

On multivariable Cox proportional hazard analysis, age (HR 1.05, 95% CI 1.01–1.10, *P* = 0.008) and HFrEF non‐responders (HR 12.33, 95% CI 2.45–61.87, *P* = 0.002) remained associated with the primary endpoint (Table 2).

**Table 2:** Univariate and multivariate associations with primary endpoint (death, cardiac transplantation or acute heart failure admission).

	Univariate associations with primary endpoint		Multivariate associations with primary endpoint	
	HR (95% CI)	*P* value	HR (95% CI)	*P* value
Age	1.041 (1.002–1.083)	0.040	1.054 (1.014–1.097)	0.008
Gender	1.436 (0.579–3.563)	0.435	1.436 (0.560–3.685)	0.451
LVEDVi	1.008 (0.998–1.018)	0.106		
LVESVi	1.007 (0.996–1.018)	0.233		
LBBB	0.709 (0.320–1.569)	0.396		
RBBB	1.378 (0.597–3.181)	0.452		
QRS duration	0.980 (0.957–1.003)	0.088		
Prior VT	0.447 (0.148–1.349)	0.153		
Prior AVB	0.259 (0.115–0.580)	0.001	0.467 (0.194–1.126)	0.090
Beta‐blocker	0.671 (0.267–1.685)	0.395		
ACE inhibitor	0.725 (0.333–1.580)	0.418		
ACE, ARB or ARNI	1.261 (0.167–9.5000)	0.822		
Device upgrade	0.518 (0.225–1.196)	0.123		
SUV_max_ > 2.5	1.153 (0.493–2.701)	0.742		
LVEF ≤ 40 responder	4.946 (1.123–21.790)	0.035	3.697 (0.810–16.873)	0.091
LVEF ≤ 40 non‐responder	15.150 (3.342–68.682)	<0.001	12.330 (2.457–61.870)	0.002

Abbreviations: ACE, angiotensin‐converting enzyme; ARB, angiotensin receptor blocker; ARNI, angiotensin receptor–neprilysin inhibitor; AVB, atrioventricular block; CI, confidence interval; HR, hazard ratio; LBBB, left bundle branch block; LVEDVi, left ventricular end‐diastolic volume index; LVEF, left ventricular ejection fraction; LVESVi, left ventricular end‐systolic volume index; RBBB, right bundle branch block; SUV_max_, maximum standardized uptake value; VT, ventricular tachycardia.

The secondary endpoint of VAEs was reached by 36 patients during follow‐up [HFrEF, *n* = 27 (42.9%); HFmrEF, *n* = 3 (17.6%); and HFpEF, *n* = 6 (30.0%); log‐rank *P* = 0.286 (Figure 2B)]. HFrEF non‐responders had significantly lower event‐free survival compared with HFrEF responders, as well as both HFmrEF and HFpEF patients, for both the primary (log‐rank *P* < 0.001) and secondary (log‐rank *P* = 0.001) endpoints. HFrEF CRT responders still had worse long‐term outcomes than CS patients with HFmrEF/HFpEF (Figure 2C,D).

## Discussion

This study assessed the cardiac functional and prognostic value of CRT response in a large contemporary population of CS patients. It also compared the prognosis of CS patients with CRT and significant LV systolic dysfunction against their counterparts without significant LV dysfunction. The main findings are as follows: (i) a significant proportion (70.5%) of CS patients with baseline HFrEF (LVEF ≤ 40%) demonstrated >5% LVEF recovery and cardiac reverse remodelling at least 6 months after CRT implantation; (ii) these HFrEF CRT responders had significantly better long‐term outcomes than HFrEF CRT non‐responders; and (iii) despite LV functional recovery at short‐term, HFrEF CRT responders still had worse long‐term outcomes than CS patients with HFmrEF/HFpEF. Age and HFrEF CRT non‐responders were associated with the primary composite endpoint on multivariable analysis.

### CRT in CS—To recover or not to recover

Prior studies investigating the effects of CRT on CS patients have generated mixed results^14^, ^15^, ^16^, ^17^, ^18^, ^19^; whilst some reported overall improvements in LV systolic function,^17^ others did not.^14^, ^15^, ^16^, ^18^ With the largest cohort of CS patients with CRT to date, we observed that over two thirds of CS patients with baseline HFrEF responded to CRT in terms of LV functional improvement and reverse cardiac remodelling. Most of the patients with HFrEF were already established on good guideline‐directed heart failure medical therapy,^12^ suggesting that the escalation to CRT implantation took place at an appropriate stage—when LV systolic dysfunction was refractory to heart failure medical therapy.^12^ This helped to better reflect a true increase in LVEF as a result of CRT, rather than being confounded by the simultaneous introduction of medical therapy.

Over half of CS patients with HFrEF had demonstrable myocardial inflammation (defined as SUV_max_ > 2.5) at baseline. Despite this, it is notable that the LV functional improvement took place after CRT. Further work is required to elucidate the mechanistic effect of CRT on cardiomyocyte remodelling in CS in the presence of myocardial inflammation, such as effects on ryanodine reception expression and sarcoplasmic reticulum function on a molecular level.^24^

### CRT and CS—Recovery is better than no recovery

The better long‐term prognosis in HFrEF CRT responders, as compared with non‐responders, appears in line with existing evidence showing the beneficial effects of CRT in other cardiac diseases.^12^ The percentage of CS patients who did not respond to CRT (~30%) is comparable with reported evidence in other heart failure aetiologies.^25^, ^26^ A large proportion of CS patients had left bundle branch morphology with wide QRS duration, which is known to respond better than other ECG morphologies.^27^

CS patients with HFpEF at baseline did not demonstrate any primary endpoints in the study but had similar event‐free survival for ventricular arrhythmia endpoints compared with patients with HFmrEF/HFrEF. It remains unclear whether CRT has exerted a protective effect in these patients or if HFpEF CS patients are an intrinsically lower risk group. The recent consensus statement on the management of CS indicated that CS patients with preserved LV systolic function and complete heart block with RV pacing may benefit more from CRT‐D than ICD.^21^ Our data do appear to support this notion from a long‐term outcome perspective. As this is the first study to compare the prognosis of CS patients with CRT in different LVEF ranges, further studies are required to better understand the underlying mechanisms of the effect of CRT in CS patients with HFpEF.

The observation of a potential association between high‐grade AVB history and better prognosis in CS patients with CRT in univariate analysis deserves further attention. It may suggest a less aggressive disease presentation in these CS patients compared with patients manifesting with severe heart failure and/or ventricular arrhythmias.^28^, ^29^ Age and accumulation of multiple cardiac complications on presentation also confer a worse prognosis than a presentation with a single complication alone.^29^ Further, CRT may confer additional benefits in patients with AVB and heart failure than those with heart failure alone without brady‐pacing indications. These hypotheses require further investigation.

### CRT and CS prognosis—Recovery is not enough

Previous evidence has demonstrated a poor clinical outcome in CS patients who underwent CRT.^14^, ^15^, ^16^, ^17^, ^18^ Our study further demonstrated that despite LV functional recovery at around 6 months, CS HFrEF CRT responders still had worse long‐term clinical outcomes compared with their counterparts with higher LVEF at baseline. The exact mechanism underlying this observation is unclear and may be multi‐faceted.

Firstly, HFrEF patients are known to carry a worse prognosis compared with patients with HFmrEF and HFpEF in other aetiologies,^12^ which may have an extrapolatory effect in CS patients with heart failure. Secondly, LV functional recovery after CRT at 6 months may not be maintained later in the clinical follow‐up, which could have significant prognostic implications.^12^, ^30^, ^31^, ^32^ Whilst the increase in LVEF over time is associated with improvement in long‐term outcomes, the converse is true for a decline in LVEF.^12^, ^30^ The transition from HFmrEF down to HFrEF confers a worse prognosis than remaining in HFmrEF.^12^, ^31^ In this study, we were only able to assess the initial LV function recovery at 6 months. A further longitudinal study is required to prospectively track the changes in LV function and correlate these changes to the eventual prognosis. Thirdly, the greater prevalence of ventricular arrhythmia events in HFrEF CRT non‐responders, as compared with both HFrEF CRT responders and HFmrEF/HFpEF patients, suggests the presence of a greater degree of myocardial fibrosis or inflammation in HFrEF CRT non‐responders.^33^ However, this hypothesis requires further investigation, with serial short‐term and long‐term follow‐ups using FDG‐PET and cardiac magnetic resonance (CMR) in CS patients with CRT in situ.

Overall, the study findings suggest that CRT can have short‐term beneficial effects on cardiac remodelling in many CS patients with LV systolic dysfunction. However, HFrEF at baseline remains a major predictor of adverse outcomes in CS, regardless of LV functional recovery with CRT. The findings potentially reinforce the importance of medical therapy to optimize cardiac function prior to CRT implantation, which requires further investigation. This study paves the way for a larger and multi‐centred study to validate the findings and further elucidate the precise mechanisms underlying the observations.

### Limitations and future directions

As a retrospective single‐centre study, the results are naturally prone to sampling bias. However, to our knowledge, this is the largest study investigating the effect of CRT on CS patients to date. Further multi‐centre registries and prospective studies are required to validate the study findings. Due to the presence of cardiac devices in all patients in the study, a significant proportion did not have baseline CMR scans or were too dated to maintain an appropriate temporal relationship with CRT implantations in the data analysis. Further, device‐related artefacts have adversely affected many patients' CMR images, despite measures being employed to minimize artefacts during scanning. This meant that meaningful analysis of late gadolinium enhancement (LGE) burden was not possible in this study to assess the degree of myocardial fibrosis and its contribution to the study results. This is a key question to be addressed in future studies ongoing in our group. Echocardiography was performed for clinical reasons and was not systematically available at long‐term follow‐up. Therefore, we did not have long‐term echocardiographic data to assess its relationship with long‐term outcomes in this CS patient cohort, which deserves further investigation. CS is also known to be a heterogeneous condition demonstrating racial disparities, and as such, CRT response in different ethnicities may defer, which is a limitation of our cohort study of predominantly white ancestry patients.^34^, ^35^, ^36^

## Conclusions

In CS patients with HFrEF (LVEF ≤ 40%), despite short‐term LV functional recovery with CRT, their long‐term prognosis remained worse than their counterparts with better baseline LV function (LVEF > 40%). This effect is not entirely driven by ventricular arrhythmia events. CS patients who were older or had HFrEF without short‐term recovery had an adverse prognosis. Larger, multi‐centre studies are required to validate these findings.

## Conflict of interest statement

The authors have none to declare.
